# Xerostomia in survivors of severe COVID-19: findings from a Latin American cohort

**DOI:** 10.3389/froh.2025.1633542

**Published:** 2025-10-07

**Authors:** Paola Andrea Escobar Villegas, Brayan Daniel Cordoba-Melo, Juan Pablo Arango-Ibanez, Maria Camila Naranjo-Ramirez, Mario Miguel Barbosa, Andrés Felipe Casanova Rojas, Andrés Fernando Mina Sánchez, Cesar José Herrera, Miguel Ángel Quintana Da Silva, Andrés Felipe Buitrago Sandoval, María Lorena Coronel Gilio, Freddy Pow Chon Long, Liliana Cárdenas Aldaz, Juan Esteban Gomez-Mesa

**Affiliations:** 1 Colegio Odontológico, Institución Universitaria Colegios de Colombia—Unicoc, Cali, Colombia; 2 Centro de Investigaciones Clínicas, Fundación Valle del Lili, Cali, Colombia; 3 Departamento de Cardiología, Fundación Valle del Lili, Cali, Colombia; 4 Departamento de Cardiología, Centros de Diagnóstico y Medicina Avanzada y de Conferencias Médicas y Telemedicina (CEDIMAT), Santo Domingo, República Dominicana; 5 Departamento de Cardiología, Instituto Cardiovascular Sanatorio MIGONE, Asunción, Paraguay; 6 Departamento de Cardiología, Fundación Santa Fe, Bogotá, Colombia; 7 Departamento de Cardiología, Instituto de Cardiología J. F. Cabral, Corrientes, Argentina; 8 Departamento de Cardiología, Hospital Luis Vernaza, Guayaquil, Ecuador; 9 Departamento de Cardiología, Hospital Eugenio Espejo, Quito, Ecuador; 10 Facultad de Ciencias de la Salud, Universidad Icesi, Cali, Colombia

**Keywords:** long COVID, xerostomia, COVID-19 sequelae, dry mouth, mouth dryness

## Abstract

**Objectives:**

SARS-CoV-2 primary affects the respiratory tract; however, evidence suggests the oral cavity can be involved in severe COVID-19 survivors. This study investigates factors associated with xerostomia in severe COVID-19 survivors from a Latin American cohort.

**Materials and methods:**

A prospective multicenter study from the Latin American Registry of Cardiovascular Disease and COVID-19, analyzed data on 272 severe COVID-19 patients from 7 institutions in 5 countries (Colombia, Dominican Republic, Ecuador, Argentina, and Paraguay). Long-term follow-up assessed demographics characteristics, comorbidities, lifestyle, cardiovascular complications, and oral health. Logistic regression in R software identified factors associated with xerostomia.

**Results:**

Xerostomia was reported in 20.6% of patients. Among affected individuals, 53.6% were female, while women represented 35.6% of those without the condition. In the overall cohort, the most common comorbidities were overweight/obesity (57.0%), hypertension (55.9%), and dyslipidemia (32.0%). Patients with xerostomia had higher rates of dyslipidemia (48.2% vs. 27.8%) and asthma/COPD (16.1% vs. 4.2%) compared to the group without xerostomia. In multivariable logistic regression, asthma/COPD (aOR: 5.14; 95% CI: 1.76–15.7), palpitations (aOR: 2.47; 95% CI: 1.04–5.94), and chest pain (aOR: 3.74; 95% CI: 1.67–8.43) were independently associated with xerostomia. Conversely, male sex was associated with lower odds of reporting xerostomia (aOR: 0.47; 95% CI: 0.24–0.89).

**Conclusion:**

These findings underscore the need for clinicians to actively assess oral health symptoms such as xerostomia in post-COVID care, particularly in patients with cardiopulmonary comorbidities and persistent systemic symptoms.

## 1 Introduction

Xerostomia (dry mouth) is common and can substantially impair quality of life, causing difficulty swallowing, reduced taste, cough, and voice changes (1). In the general population, medication use is the leading cause: more than 400 prescription and over-the-counter drugs are associated with reduced salivary flow and xerostomia (e.g., antihypertensives, antidepressants, anticholinergics, diuretics, opioids, nonsteroidal anti-inflammatory drugs, antihistamines) (2–5). Less commonly, xerostomia arises from systemic conditions such as Sjögren's syndrome, connective tissue diseases, diabetes mellitus, chronic kidney disease, and autoimmune disorders, as well as infections like human immunodeficiency virus and cytomegalovirus (2, 6).

While the primary manifestation of SARS-CoV-2 infection in humans is in the respiratory tract, it raises the question of whether this virus, like other infections, can affect the oral cavity. It has been demonstrated that salivary glands are a potential target for COVID-19 infection due to the documented expression of the ACE2/transmembrane serine protease 2 receptor in the epithelial cells of salivary glands (7, 8). Emerging evidence suggests that the oral cavity is a potential target of COVID-19, with persistent alterations observed in a majority of survivors well beyond their clinical recovery. Individuals who have recovered from COVID-19 have reported various clinical conditions in the upper airway, including xerostomia, increased thirst, and persistent dry cough, which endure for an extended post-COVID period and can be defined as long COVID or post COVID-19 syndrome (9).

Evaluating long-term oral complications in COVID-19 patients is crucial due to the wide variability in prevalence depending on the study and methodology employed. A literature review included seven studies on xerostomia, involving a total of 654 COVID-19 survivors from Italy, Turkey, China, India, Israel, and Colombia. Reported prevalence ranged from 2% to 40% with follow-up of 28–230 days; the review did not distinguish by hospitalization status or disease severity (10).

Further research is needed to clarify the long-term impact of SARS-CoV-2 infection on oral health, as current evidence is scarce and heterogeneous. Important gaps remain regarding the relative contribution of viral effects, systemic inflammation, neurological mechanisms, and treatments such as antibiotics to the development of xerostomia and other oral manifestations (11). Addressing these uncertainties requires studies that document the occurrence of xerostomia and explore its associations in well-defined patient populations.

## 2 Objective

To describe the occurrence and associations of xerostomia in a cohort of Latin American survivors of severe COVID-19.

## 3 Methods

### 3.1 Study design and participants

A prospective cohort study was conducted using data from the CARDIO COVID 19–20 Registry (Registro Latinoamericano de Enfermedad Cardiovascular y COVID-19) and its long-term extension, the CARDIO COVID 20–21 registry. The CARDIO COVID 19–20 registry was an observational, multicenter, ambispective, and hospital-based registry of patients with confirmed COVID-19 infection who required in-hospital treatment in Latin America, while the CARDIO COVID 20–21 registry prospectively followed a subset of these patients after discharge**.** The CARDIO COVID 19–20 registry enrolled patients over 18 years old hospitalized for more than 24 h with confirmed SARS-CoV-2 infection vía RT-PCR following World Health Organization (WHO) guidelines. The study was conducted across 44 hospitals in 14 Latin American countries, including Argentina, Brazil, Chile, Colombia, Costa Rica, Dominican Republic, Ecuador, El Salvador, Guatemala, Mexico, Panama, Paraguay, Peru, and Venezuela (12).

Between June 2020 and June 2021, a total of 3,260 patients were enrolled in the CARDIO COVID 19–20 registry. Of these, 869 (26.7%) died during hospitalization and 417 (12.8%) were excluded because they did not fulfill severity criteria, leaving 1,974 (60.5%) patients classified as severe COVID-19. This population served as the source for long-term follow-up in the CARDIO COVID 20–21 registry.

The criteria for a severe COVID-19 episode were defined as having at least one of the following: high risk of venous thromboembolism (elevated D-dimer: 1219 patients, 61.8%), the necessity for intensive care unit (ICU) admission (896 patients, 45.4%), cardiovascular complications during hospitalization (such as arrhythmia, arterial or venous embolism, coronary events, and heart failure: 406 patients, 20.6%), or myocardial injury (elevated troponin levels above the 99th percentile: 302 patients, 15.3%).

At 30-day follow-up, 318 unreachable patients and 37 deaths left 1619 (49.7%) severe COVID-19 patients for long-term follow-up. Only seven of 44 institutions (Colombia, Dominican Republic, Ecuador, Argentina, and Paraguay) joined the second registry (CARDIO COVID 20–21), excluding 1105 patients. Of the remaining 514 patients, 242 were lost, leaving a final cohort of 272 patients.

Among the 272 severe COVID-19 patients included in the long-term follow-up, 39.3% (107/272) met one severity criterion, 35.7% (97/272) met two, 24.3% (66/272) met three, and 0.7% (2/272) met all four. Within these strata, xerostomia was present in 23.4% (25/107), 20.6% (20/97), 15.2% (10/66), and 50.0% (1/2), respectively.

**Figure 1:**
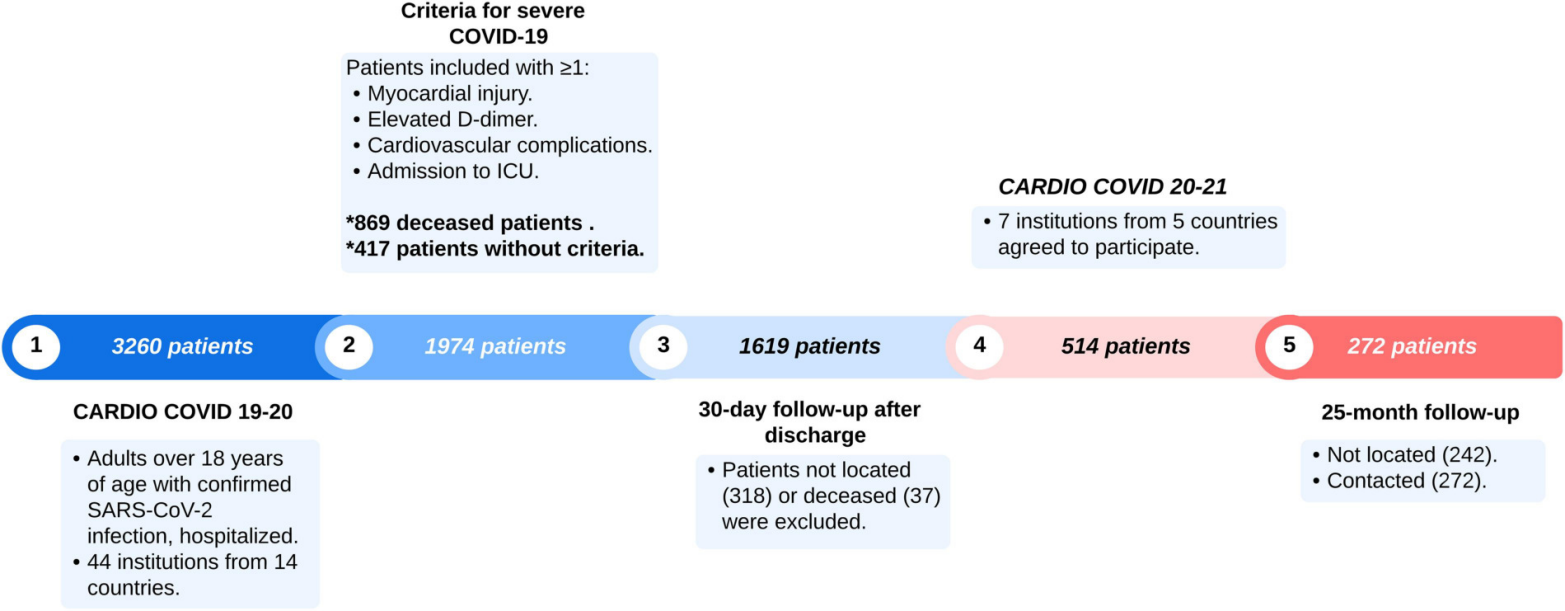
Patient selection flowchart.

Follow-up visits occurred at a median of 25 months post-discharge, in person (192 patients) or via telephone (80 patients). Under physician supervision, self-reported symptoms and medical histories and assessments for anxiety, depression, stress, quality of life, and cognitive impairment were collected. Figure 1 summarizes the patient selection and exclusion process, providing the study's design and participant flow. Oral health symptoms were assessed using a standardized, physician-supervised, self-report questionnaire. For xerostomia, patients were directly asked: “Have you experienced a persistent dry mouth or lack of saliva over the past month?” The full set of questions and guidance for healthcare professionals is available in the Supplementary Table S1.

Both the CARDIO COVID 19–20 and CARDIO COVID 20–21 registries were coordinated by the Inter-American Council of Heart Failure and Pulmonary Hypertension (CIFACAH) of the Inter-American Society of Cardiology (IASC) with operational support from the Clinical Research Center at Fundación Valle del Lili (FVL) in Cali, Colombia. All patients provided written informed consent, and data was securely stored and accessed only by the principal investigators. The study received ethical approval from the Academic Committee of the IASC and the Institutional Review Board (IRB) of Fundación Valle de Lili (2021.1756), following the ethical principles of the 1975 Declaration of Helsinki.

### 3.2 Statistical analysis

Categorical variables were evaluated by assessing their frequency and percentage, whereas continous variables were studied by analyzing their median with interquartile range [interquartile range (IQR)]. Cross-tabulations with Chi-square or Fisher's exact tests were used to compare proportions between groups. The Kruskal–Wallis and Mann–Whitney U tests were used to compare continuous variables between groups. Logistic regression analyses, both univariate and multivariate, were performed to examine the contribution of specific variables to the development of xerostomia. Variables with a *P* value <0.05 in the univariate analysis, as well as those reported in the literature as potential covariates or considered clinically significant, were included in the multivariate analysis. All analyses were performed using R statistical software (version 4.02). The REDCap (Research Electronic Data Capture) electronic database system was used to collect data from the medical records.

## 4 Results

Table 1 summarizes the baseline characteristics of the study population. Overall, the median age was 58 years, without differences between patients with and without xerostomia. A higher proportion of women reported xerostomia than men (53.6% vs. 35.6%; *P* = 0.021). Dyslipidemia (48.2% vs. 27.8%; *P* = 0.006) and asthma/COPD (16.1% vs. 4.2%; *P* = 0.004) were also significantly more frequent in the xerostomia group, whereas the remaining comorbidities and pharmacological treatments showed comparable distributions.

**Table 1:** Xerostomia by demographic and clinical characteristics.

Characteristic	Overall (*N* = 272)^a^	Xerostomia: Yes (*n* = 56)^a^	Xerostomia: No (*n* = 216)^a^	*P*-value
Age, median (IQR)	58 (48–69)	57 (47–63)	58 (48–69)	0.4^b^
Sex				
Male	165 (60.7)	26 (46.4)	139 (64.4)	**0** **.** **021** ^c^
Female	107 (39.3)	30 (53.6)	77 (35.6)	
Comorbidities				
Overweight/Obesity	155 (57.0)	35 (62.5)	120 (55.6)	0.4^c^
Hypertension	152 (55.9)	33 (58.9)	120 (55.6)	0.8^c^
Dyslipidemia	87 (32.0)	27 (48.2)	60 (27.8)	**0**.**006**^c^
Diabetes mellitus	79 (29.0)	22 (39.3)	57 (26.4)	0.069^c^
CKD	39 (14.3)	9 (16.1)	30 (13.9)	0.6^c^
Asthma/COPD	18 (6.6)	9 (16.1)	9 (4.2)	**0**.**004**^c^
Pharmacological therapy				
ARBs	83 (30.5)	23 (41.1)	60 (27.8)	0.072^c^
Statins	68 (25.0)	15 (27.0)	53 (24.5)	0.7^c^
Hypoglycemic agents	45 (16.5)	11 (19.6)	34 (15.7)	0.6^c^
CCBs	40 (14.7)	9 (16.1)	31 (14.4)	0.8^c^
BB	36 (13.2)	9 (16.1)	27 (12.5)	0.5^c^
Diuretics	36 (13.2)	10 (17.9)	26 (12.0)	0.3^c^
ACE inhibitors	27 (9.9)	5 (8.9)	22 (10.2)	>0.9^c^

ACE, angiotensin-converting enzyme; ARBs, angiotensin receptor blockers; BB, beta blockers; CCBs, calcium channel blockers; CKD, chronic kidney disease; COPD, chronic obstructive pulmonary disease.

Bolded *P*-values indicate statistical significance.

a Values are expressed as median (interquartile range) or *n* (%).

b *P*-value from Wilcoxon rank sum test.

c *P*-value from Fisher's exact test.

Vital signs and anthropometry at the 25-month in-person evaluation (192/272 patients, 70.6%) are shown in Table 2. In this subset, no statistically significant differences were observed between patients with and without xerostomia in respiratory rate, heart rate, systolic or diastolic blood pressure, oxygen saturation, or body mass index.

**Table 2:** Vital signs and anthropometry at 25-month follow-up (patients with face-to-face interview).

Characteristic	Overall (*N* = 192)^a^	Xerostomia: Yes (*n* = 53)^a^	Xerostomia: No (*n* = 139)^a^	*P*-value^b^
Vital signs				
Respiratory rate^c^	17 (17, 18)	17 (17, 18)	17 (16, 18)	0.7831
Heart rate^d^	72 (66, 80)	72 (65, 78)	71 (66, 80)	0.5242
SBP^e^	126 (116, 138)	125 (113, 137)	127 (116.5, 138)	0.6513
DBP^e^	80 (70, 84)	80 (67, 85)	80 (71, 84)	0.8250
Oxygen saturation^f^	97 (96, 98)	97 (96, 98)	97 (95.5, 98)	0.5775
Anthropometry				
BMI^g^	27.9 (25.1, 31.2)	27.2 (25.2, 31.5)	28.1 (24.95, 31.2)	0.8639

BMI, body mass index; bpm, beats per minute; DBP, diastolic blood pressure; rpm, respirations per minute; SBP, systolic blood pressure.

a Values are expressed as median (interquartile range).

b *P*-value from Wilcoxon rank sum test.

c Units: respirations per minute.

d Units: beats per minute.

e Units: millimeters of mercury.

f Units: percentage.

g Units: kilograms per square meter.

At the 25-month follow-up, functional status, oral manifestations, and systemic symptoms were assessed in the cohort (*n* = 272), as detailed in Table 3. Patients with xerostomia were less frequently classified as NYHA I than those without xerostomia (48.2% vs. 69.0%; *P* = 0.006), while classes II and III were more common in the xerostomia group. Dysphagia (30.4% vs. 7.9%; *P* < 0.001), salivary gland pain/swelling (7.1% vs. 1.4%; *P* = 0.035), and submandibular swelling (5.4% vs. 0.5%; *P* = 0.028) were also more frequent among patients with xerostomia, whereas other oral findings did not differ significantly. Systemic symptoms, including fatigue (75.0% vs. 46.3%; *P* < 0.001), myalgia/arthralgia (76.8% vs. 31.9%; *P* < 0.001), palpitations (50.0% vs. 15.3%; *P* < 0.001), and chest pain (51.8% vs. 13.9%; *P* < 0.001), were likewise more common in the xerostomia group, while dyspnea did not show a statistically significant difference (*P* = 0.056).

**Table 3:** Functional status, oral manifestations, and systemic symptoms at 25-month follow-up.

Characteristic	Overall (*N* = 272)^a^	Xerostomia: Yes (*n* = 56)^a^	Xerostomia: No (*n* = 216)^a^	*P*-value
NYHA functional class				
Class I	176 (64.7%)	27 (48.2%)	149 (69.0%)	**0** **.** **006** ^b^
Class II	63 (23.2%)	17 (30.4%)	46 (21.3%)	
Class III	31 (11.4%)	12 (21.4%)	19 (8.8%)	
Class IV	2 (0.7%)	0 (0%)	2 (0.9%)	
Oral manifestations				
Dysphagia	34 (12.5%)	17 (30.4%)	17 (7.9%)	**<0**.**001**^c^
Oral ulcers^d^	11 (4.0%)	4 (7.1%)	7 (3.2%)	0.2^c^
Salivary gland pain/swelling^e^	7 (2.6%)	4 (7.1%)	3 (1.4%)	**0**.**035**^c^
Gum bleedin^g^	6 (2.2%)	2 (3.6%)	4 (1.9%)	0.6^c^
Oral lesions^f^	4 (1.5%)	1 (1.8%)	3 (1.4%)	>0.9^c^
Submandibular swelling^g^	4 (1.5%)	3 (5.4%)	1 (0.5%)	**0**.**028**^c^
Burning mouth/tongue^h^	3 (1.1%)	1 (1.8%)	2 (0.9%)	0.5^c^
Tongue redness	3 (1.1%)	2 (3.6%)	1 (0.5%)	0.11^c^
Signs and symptoms				
Fatigue	142 (52.2%)	42 (75.0%)	100 (46.3%)	**<0**.**001**^c^
Myalgia/arthralgia	112 (41.2%)	43 (76.8%)	69 (31.9%)	**<0**.**001**^c^
Dyspnea	68 (25.0%)	20 (35.7%)	48 (22.2%)	0.056^c^
Palpitations	61 (22.4%)	28 (50.0%)	33 (15.3%)	**<0**.**001**^c^
Chest pain	59 (21.7%)	29 (51.8%)	30 (13.9%)	**<0**.**001**^c^

Bolded *P*-values indicate statistical significance.

a Values are expressed as *n* (%).

b *P*-values from Fisher's exact test with simulated *p*-value (2,000 replicates), used for NYHA functional class (ordinal variable with 4 categories).

c *P*-values from Fisher's exact test (categorical dichotomous variables).

d Oral ulcers: lesions inside the mouth.

e Salivary gland pain/swelling: pain or swelling in the salivary glands (in front of the ear or cheek).

f Oral lesions: spots or lesions on the lips or inside the mouth.

g Submandibular swelling: swelling under the jaw.

h Burning mouth/tongue: burning sensation in the mouth or tongue.

**Figure 2:**
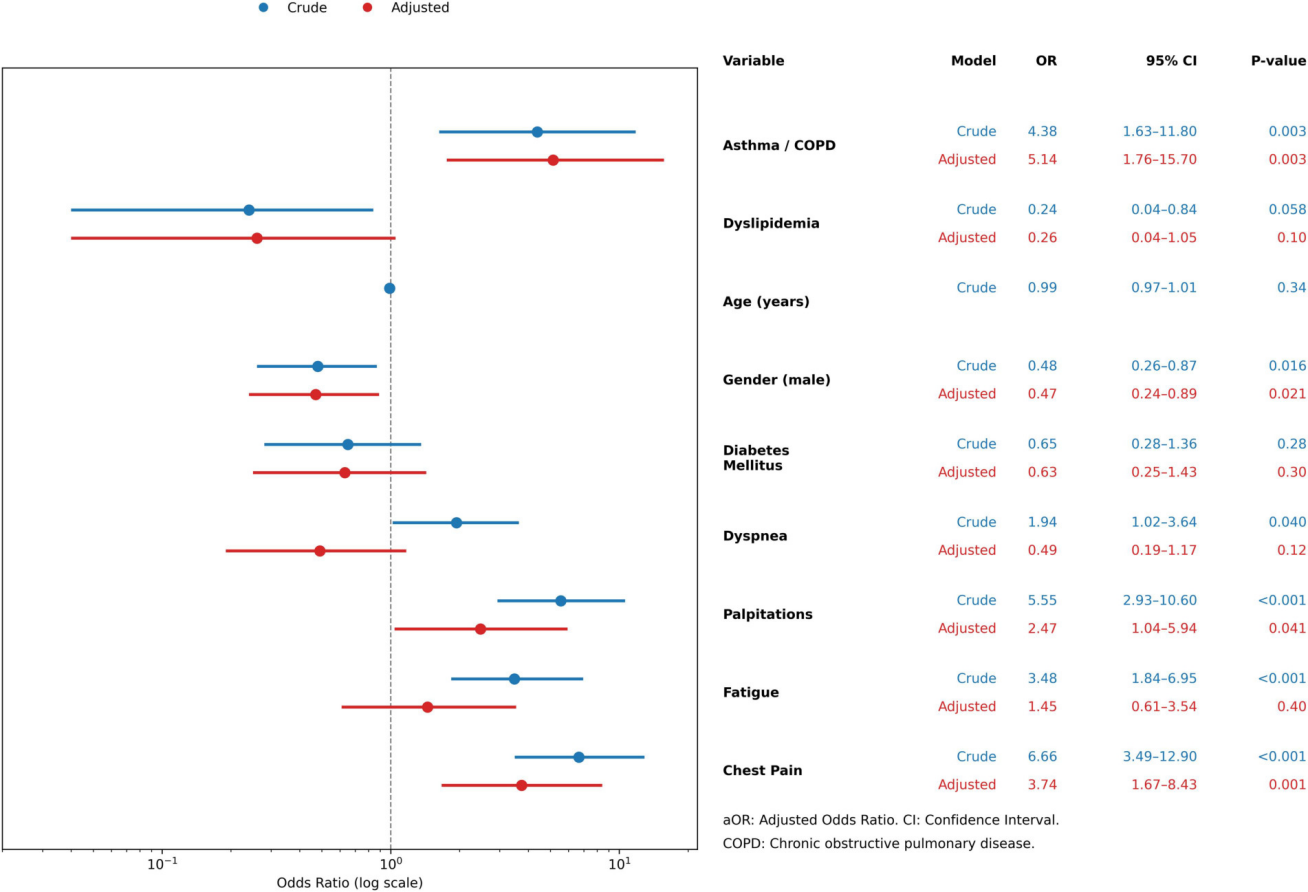
Summary of logistic regression models for xerostomia.

Figure 2 summarizes the univariable and multivariable logistic regression analyses for factors associated with xerostomia. In the adjusted model, male sex was associated with lower odds of xerostomia (*aOR*: 0.47; 95% CI: 0.24–0.89; *P* = 0.021), while asthma/COPD was associated with higher odds (*aOR*: 5.14; 95% CI: 1.76–15.7; *P* = 0.003). Among symptoms, palpitations (*aOR*: 2.47; 95% CI: 1.04–5.94; *P* = 0.041) and chest pain (*aOR*: 3.74; 95% CI: 1.67–8.43; *P* = 0.001) remained significantly associated with xerostomia. Dyslipidemia, diabetes, age, dyspnea, and fatigue did not show significant associations after adjustment, although dyspnea and fatigue were significant in univariable analyses.

## 5 Discussion

This study analyzed 272 survivors of severe COVID-19 from five Latin American countries and found that xerostomia persisted in approximately one-fifth of patients after more than two years of follow-up. The condition was more frequent in women and independently associated with asthma/COPD, palpitations, and chest pain, underscoring its multifactorial nature.

Strengths of this study include its multicenter design across different countries, the use of in-person assessments in most participants, and a follow-up period exceeding two years, which is longer than most published cohorts. These aspects enhance representativeness and external validity. Nonetheless, several limitations must be acknowledged. The retrospective design may introduce selection bias, xerostomia was self-reported without objective validation, baseline oral health status was not available, and the analysis of medications was not sufficiently detailed to establish their contribution. These elements restrict causal interpretation and should be considered when attempting to generalise from the findings.

Our findings are consistent with previous studies reporting a high burden of comorbidities among severe COVID-19 survivors (13, 14). Compared to prior cohorts, the prevalence of xerostomia in our population (20.6%) was lower than in studies from Italy (30%) and Colombia (26%) (11, 15), likely reflecting differences in follow-up duration and patient selection. Similar heterogeneity has been documented, with reported prevalences ranging from 2% to 40% depending on study design and methodology (10). Importantly, the association with asthma/COPD persisted beyond two years, extending prior evidence from shorter follow-up studies and suggesting that underlying respiratory disease and its treatments remain important determinants (11, 16).

Other oral manifestations were also less frequent than in international reports. Dysphagia occurred in 12.5% of patients, lower than the pooled prevalence reported in systematic reviews (17, 18). Similarly, oral ulcers were uncommon in our cohort compared to previous literature (11, 19). These differences likely arise from variability in populations studied, the tools used to capture oral symptoms, and the timing of follow-up. Together, these observations underscore the need for standardized methods to assess oral sequelae in COVID-19 survivors.

Pharmacological therapies may further contribute to xerostomia. More than 400 prescription and over-the-counter medications are recognized causes of reduced salivary flow and dry mouth in the general population (e.g., antihypertensives, antidepressants, anticholinergics, diuretics, opioids, nonsteroidal anti-inflammatory drugs, antihistamines) (4, 5). Several drug classes commonly prescribed in COVID-19 survivors, including ACE inhibitors, beta blockers, calcium channel blockers, diuretics, and ARBs, have been specifically linked to salivary dysfunction (20, 21). In our cohort, ARBs were more frequent among patients with xerostomia, although without statistical significance. This highlights the challenge of disentangling the contributions of pharmacotherapy, comorbid conditions, and COVID-19 sequelae to the persistence of oral dryness.

Taken together, these findings indicate that xerostomia in long COVID is multifactorial, likely resulting from interactions between systemic comorbidities, chronic pharmacological treatment, and potential sequelae of SARS-CoV-2 infection (7, 11, 20, 21). Clinically, this emphasizes the importance of incorporating oral health assessment into long-term follow-up of COVID-19 survivors. Physicians and dental practitioners should recognize its impact on quality of life and consider multidisciplinary approaches (8, 9). Future research should prioritize objective assessments of salivary function, detailed evaluation of medication exposures, and the identification of modifiable risk factors, with the goal of guiding preventive and therapeutic strategies.

## 6 Conclusions

These findings underscore xerostomia as a relatively common symptom in survivors of severe COVID-19, highlighting the need for increased awareness among physicians and dental health practitioners to actively screen for and manage this condition during long-term follow-up. Given the association of xerostomia with systemic conditions such as asthma/COPD, dyslipidemia, and cardiovascular disease, a multidisciplinary approach may be essential in addressing its impact on patient well-being. Furthermore, our findings suggest that long-term evaluations are crucial to better understanding the persistence of oral symptoms in COVID-19 survivors and their potential underlying mechanisms. Future prospective studies should focus on identifying modifiable risk factors and evaluating the role of pharmacotherapy and inflammatory pathways in the development of xerostomia, ultimately guiding more effective preventive and therapeutic strategies.

## Data Availability

The data that support the findings of this study are not openly available due to reasons of sensitivity and are available from the corresponding author upon reasonable request.
