# Supplementary material for: Xerostomia in survivors of severe COVID-19: findings from a Latin American cohort
Source: Front Oral Health. 2025 Oct 7;6:1633542. doi: 10.3389/froh.2025.1633542 (PMC12537734; doi:10.3389/froh.2025.1633542)
Supplement: Supplementary file 1 [file Table1.docx]

Supplementary Material

**Supplementary table 1: Oral health symptom self-report guided by a healthcare professional**

| Symptom | Question | Guidance for the Healthcare Professional |
| --- | --- | --- |
| Xerostomia (Dry mouth) | Have you experienced a persistent dry mouth or lack of saliva over the past month? | Explain that dryness can occur at any time of day or during various activities. |
| Difficulty swallowing | Have you had trouble swallowing food or liquids, as if your throat were dry, over the past month? | Ensure the patient includes both liquids and solids in their response. |
| Mouth ulcers | Have you noticed any sores or lesions inside your mouth over the past month? | Clarify the difference between an ulcer and other types of oral lesions, such as superficial sores. |
| Pain in salivary glands | Have you experienced pain or swelling in the area of the salivary glands (in front of the ear or in the cheek) over the past month? | Guide the patient to identify the salivary glands precisely, either in front of the ear or in the cheek. |
| Bleeding gums | Have you noticed your gums bleeding easily while brushing or spontaneously over the past month? | Explain that bleeding can be occasional or recurrent, and ask about oral hygiene habits. |
| Swelling under the jaw | Have you experienced swelling under the jaw over the past month? | Ensure the patient differentiates this swelling from other causes such as infections. |
| Burning sensation in mouth or tongue | Have you felt a burning or irritation sensation in your mouth or on your tongue over the past month? | Ask the patient to specify when and how frequently it occurs. |
